# Acceptability and usability of HIV self-tests in two European countries: findings from surveys of clients at non-governmental organisations in Lithuania and Italy

**DOI:** 10.1186/s12879-021-06442-8

**Published:** 2021-09-13

**Authors:** Sophie G. Nash, Manuel Maffeo, Giedrius Likatavicius, Lella Cosmaro, Kestutis Rudaitis, Aleksandras Lapsinov, Qudsia Enayat, Valerie Delpech, Meaghan Kall

**Affiliations:** 1 Blood Safety, Hepatitis, STI and HIV division, National Infection Service, Public Health England, London, UK; 2 Arcigay - Associazione LGBTI Italiana, Bologna, Italy; 3 Demetra, Vilnius, Lithuania; 4 Fondazione LILA Milano – Italian League for Fighting AIDS, Milan, Italy

**Keywords:** HIV, HIV testing, Self-testing, Survey, Acceptability, Usability

## Abstract

**Background:**

Uptake of HIV self-tests (HIVST) remains low in Europe. We conducted two separate surveys to understand facilitators and barriers to the use of HIVST in two European countries, as part of the EU INTEGRATE Joint Action. In both countries, HIV has been legal since 2016. In Lithuania, where HIVST sales have been low, the survey primarily assessed acceptability whilst in Italy, with better HIVST uptake, usability was the focus.

**Methods:**

Participants were recruited through community HIV testing sites, and in Lithuania also through social media. In Lithuania, participants self-completed a survey on their testing history, and attitudes toward and experiences with self-testing. In Italy participants performed an HIVST (Mylan Autotest) while being observed by a community health worker (CHW). Both participants and CHW completed a self-administered survey evaluating the experience of the participant.

**Results:**

In Lithuania, awareness of HIV self-testing (75%) was high among the 138 people who completed the survey. Privacy and confidentiality (70%) was the most common reason to use an HIVST whilst cost was reported as the main barrier by 60%, only 15% were willing to pay the current price. Almost half (42%) were concerned about doing the test incorrectly and 36% preferred that a trained person could discuss their result. Purchasing HIVST at a pharmacy (70%) or online (61%) was favoured and 68% would opt to simultaneously test for other infections.

In Italy, 28 people who had never used an HIVST before were observed using one. 43% found the test easy to use but CHWs reported that 36% of participants failed at least one step. The quick result (68%) was the most common reason to use one again, yet the main concerns were the lack of counselling (50%) and reading result alone (32%).

**Conclusions:**

HIVST are acceptable and usable, however cost is a major barrier. Local and national strategies are needed to increase awareness of and access to HIVST and target HIVST campaigns toward key risk groups such as MSM. Meanwhile, steps can be taken to improve testing instructions and support for self-testers. Offering multiplex testing for other infections would also likely increase uptake.

## Background

Across Western European countries (EU/EEA) the number of new HIV diagnoses has slightly declined between 2009 and 2018 (from 32,653 to 26,164), which is mostly driven by a substantial decline in a subset of countries [1]. These national declines are attributed to implementation of comprehensive combination prevention programmes in these countries. Despite this decline in new diagnosis, nearly half (48.6%) of new diagnoses in the EU/EEA in 2017 were made at a late stage of infection [2] indicating the need for easily accessible HIV testing options. Over the past decade there has been an expansion of HIV testing into non-clinical settings [3, 4] where in some countries, testing is offered by community health workers (CHW) and other testing technologies have also become available including HIV self-sampling and HIV self-testing [5, 6]. Both testing modalities have the benefit that users do not need to visit a clinic and can carry out the test at their own convenience. Self-sampling has become common in only a few countries as it relies on a reliable laboratory network [7]. Implementation of HIV self-tests (HIVST) is at varying stages in European countries. It is legal in 22 countries in Europe (69%) and 14 countries also have national policies that support its implementation. Overall, less than half (47%, 15/32) have introduced HIVST but uptake remains low [8].

The benefits and high acceptability of HIVST have been documented globally [9, 10] and it has been recommended by the WHO since 2016 [11]. There is however a lack of published studies on the acceptability and usability in the European context, particularly with regard to their use in real-life scenarios. Furthermore, as HIVST are often obtained through private companies and used anonymously, it has been difficult to monitor and evaluate their uptake, as well as to understand the contribution of HIVST in diagnosing people and the volume of users linked to appropriate services.

In Lithuania, new diagnoses rates remain low, but have risen over the past decade where two-thirds of people were diagnosed late in 2017 [2], indicating that HIV combination prevention measures require scaling up. The HIV epidemic is concentrated in people who inject drugs (PWID), men who have sex with men (MSM) and prisoners. HIV testing is available at clinics and large hospitals where key population groups, pregnant women and TB patients are all offered HIV testing. Community HIV testing is not common due to legal restrictions.

In Italy, the number of new diagnoses has declined by 26% between 2009 and 2018. But, in 2018 57% of people were diagnosed at a late stage of infection [12]. In 2019, there were an estimated 130,000 people living with HIV [13], of whom 86–91% were diagnosed [14], highlighting that further work is required to increase uptake of HIV testing. In 2019, most new diagnoses were in heterosexuals and MSM (42% respectively) and smaller proportion in PWIDs (6%) [15]. Testing is available at infectious disease units at hospitals and clinics for STIs. Community testing services are available in some cities but not yet widespread.

Both countries have HIVST included in their national HIV testing policies and HIVST was legalised and made available for private purchase in 2016. They are predominantly available in pharmacies at a similar price point in both countries (20–30€). There is anecdotal evidence that in Lithuania, HIVST have had limited uptake, there is low public awareness for their availability and they are hard to access. In Italy uptake has been higher, but during workshops related to INTEGRATE Joint Action [16], members of community non-governmental organisations (NGOs) expressed concerns that there is insufficient information to support an individual to carry out the test correctly and link to care in case of a reactive result.

Two separate surveys were used to assess the different stages of implementation and better understand barriers to the use of HIVST in Lithuania and Italy. In Lithuania the survey sought to understand awareness of, attitudes toward and barriers to the use of HIVST among the general public. The Italian survey aimed to investigate the experience of people using a HIVST for the first time, focusing on users’ feelings about performing the test, their ability to carry out the test correctly, whether they would be likely to use one again in the future and if so what support they would like.

## Methods

### Lithuania - acceptability

The survey was developed using questionnaires previously used and published in the literature that also aimed to assess acceptability of HIVST [17, 18]. The proposed questions were reviewed and tailored to fit the Lithuanian context where required. The survey was carried out in November 2019 through online survey and through face to face interviews. The inclusion criteria for participants were at least 18 years old and fluent in Lithuanian. Survey participants were recruited through social media channel used for communicating prevention messages and directly in person when they came to get tested for HIV at Demetra’s (an HIV NGO based in Lithuania) testing service. Participants were asked about their HIV risk,^1^ testing history, knowledge of HIVST, preferences and any concerns relating to using an HIVST, willingness to pay and information they would like to accompany the test. Data from the online and paper-based responses were combined.

### Italy - usability

Two complementary surveys were developed to assess the usability of HIVST by the general public. One survey was designed to be completed by individuals using an HIVST for the first time, to assess the experience and the need for additional support. The other survey was designed to be completed by an individual observing the tester, to verify that the tester could accurately complete all the required steps of the test. Participants were recruited through clients of two Italian NGOs: ARCIGAY and Fondazione LILA MILANO. Inclusion criteria for participants were at least 18 years old, fluent in Italian and never having used an HIVST before. The most common HIVST available in Italy are produced by Mylan and a limited number of HIVST were provided by the company for free for the purpose of this study. Eligible participants were asked to perform an HIVST under the supervision of a CHW. Both the users and CHWs completed their retrospective questionnaires after the HIVST was carried out.

Results from each survey were analysed and separate descriptive analyses were performed. All survey questions can be found in Appendix 1–3.

## Results

### Lithuania - acceptability

**Fig. 1:**
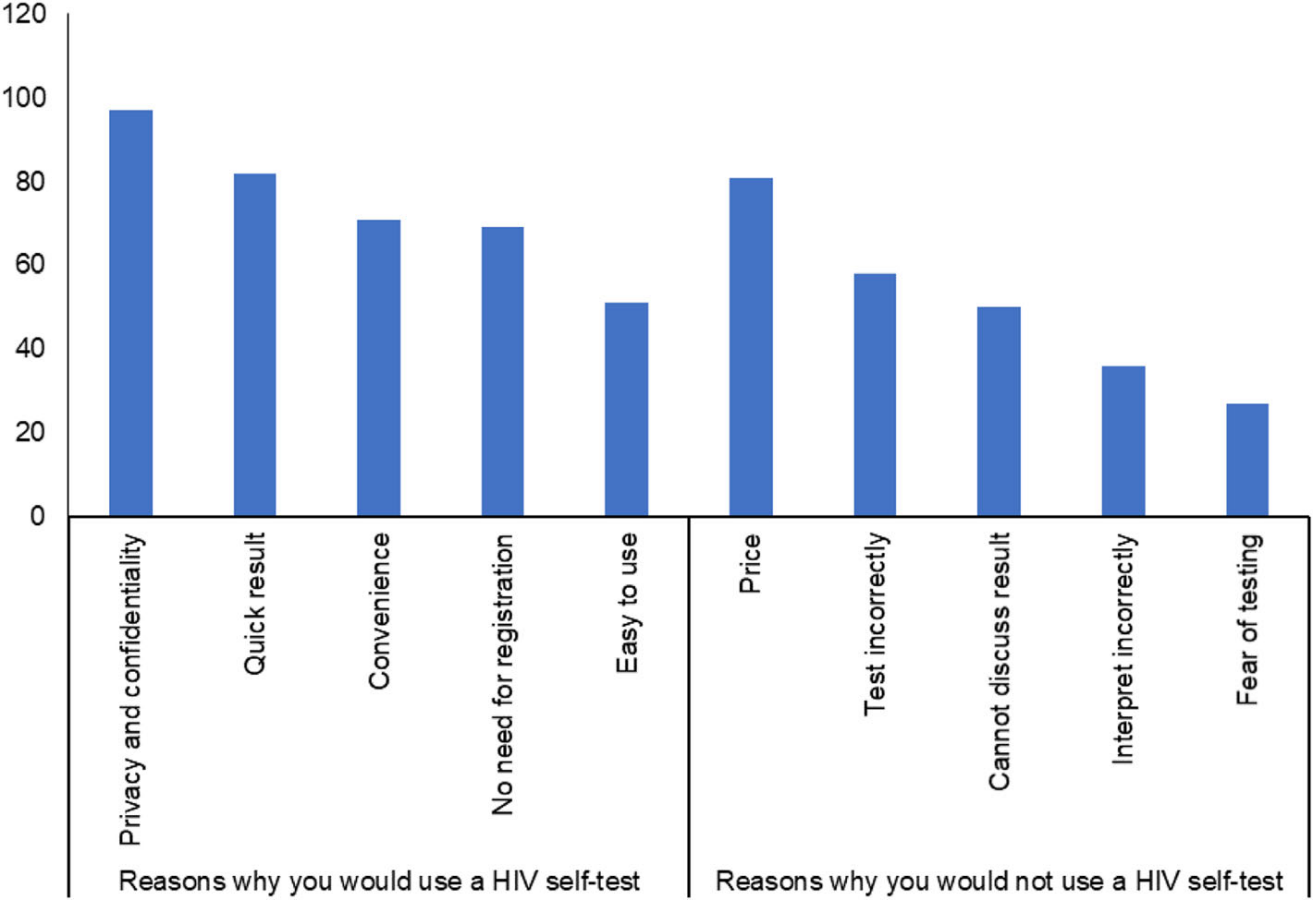
Reasons why participants would or would not use an HIV self-test in the future

In total, 138 people completed the survey (122 men and 16 women) and nearly all were educated to at least high school level (136/138). HIV risk was assessed using questions about sexual history, history of HIV indicator conditions and engagement in needle sharing practices^1^. The majority reported at least one HIV risk factor (86%, 119/138), of whom most had tested for HIV in the past year (67%, 80/119). Awareness of HIVST was relatively high among survey participants, most knew you could test for HIV using a self-test (75%, 103/138) and the majority had tested for HIV before (83%, 114/138). Further to this, most respondents (74%, 102/138) said they would likely buy and use an HIVST in the future. When respondents were asked about their preferred mode of testing, 46% (63/138) people indicated their preference for using a blood test; 22% (31/138) would opt for an HIVST and 32% (44/138) were unsure. Most people (80%, 110/138) said they would trust the result of an HIVST and would know how to proceed in case of a reactive result (66%, 91/138). The top cited reasons respondents gave for using an HIVST in the future were privacy and confidentiality, (70%, 97/138) regardless of reporting HIV factors (Fig. 1). For those who reported an HIV risk factor, the second highest reason for using an HIVST was the quick result (63%, 74/119). While for those who did not report any risk factor, it was that HIVST does not require the tester to share their personal details (64%, 12/19). However, most people did not think that HIVST would be easy to use (63%, 87/138).

The most common cited barrier to the use of HIVST was the price of the test for both those who reported an HIV risk factor (60%, 71/119) and those who did not report one (53%, 10/19). Only 16% (17/105) of participants would be willing to pay more than 20€ for the test and 15% (21/138) were currently able to pay more than 20€ for the test. Further to this, 42% (58/138) were concerned about performing the test incorrectly and a third (36%, 50/138) were concerned about having nobody with whom to discuss the test result. Most participants would prefer to purchase an HIVST at a pharmacy (70%, 96/138) or online (61%, 84/138). The majority also would like be able to test for other infections at the same time (68%, 94/138) and to have the contact details for support services (67%, 93/138).

### Italy - usability

In total, 28 people were observed using an HIVST for the first time. Most participants were men (57%, 18/28), heterosexual (61%, 17/28), all were educated to at least high school level and the average age was 36 years old. Two thirds (21/28) had tested for HIV before and a third (9/28) were not aware that you could test for HIV using a self-test.

**Fig. 2:**
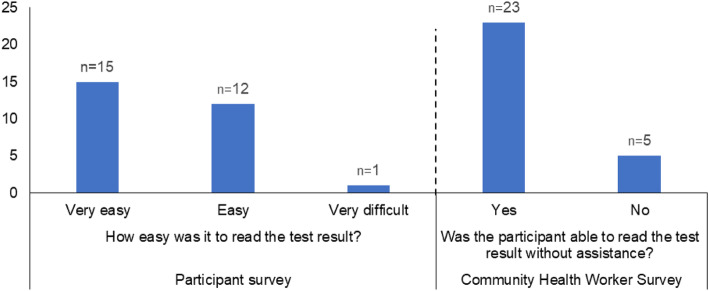
Ease of reading the test result, as reported by the participant and community health worker

Most participants were successfully able to complete the HIVST, and over a third of participants (12/28) said that it was easy or very easy. However, CHWs reported that just over a third (9/28) of participants failed at least one step when performing the HIVST or that they required assistance to complete the test. The most common difficulties reported were the collection of an adequate blood sample (7/28) and difficulties understanding how to activate the test (6/28). Further to this, CHWs reported that 10 users did not record the time while waiting for the test result (15–20 min are required). Only one participant said that it was very difficult to read the test result but the CHWs reported that five people could not read the test result without assistance (Fig. 2).

Nearly all participants reported that they thought the test was reliable (27/28) and most said that they were satisfied with their testing experience (20/28). For those who were not satisfied, most stated it was because they did not complete the test or that the instructions were difficult to understand. Most participants said they would likely use an HIVST in the future (20/28) and the main reported reasons to use an HIVST were: rapid result (68%, 19/28), no need for medical prescription (36%, 10/28) and privacy (21%, 6/28). Preference for future testing was split where nearly half (13/28) would prefer to take the test alone and the other half would prefer to take the test with the support of someone else (15/28).

The reasons reported by participants about why they would not use an HIVST in the future were: lack of counselling (50%, 14/28), worried about reading result alone (32%, 9/28) and cost (29%, 8/28). In terms of cost, nearly a fifth of participants would be willing to pay more than 20€ (5/28) and most would like to purchase the HIVST at a pharmacy (75%, 21/28). When using an HIVST in the future, participants would like to receive contact details to access: relevant NGOs (64%, 18/28), counselling (50%, 14/28), testing for other STIs (36%, 10/28).

Full breakdowns for the survey results are available in Appendix 4–6.

## Discussion

This study sought to understand facilitators and barriers to the use of HIVST in Lithuania and Italy. In Lithuania, the survey demonstrated high acceptability and demand for HIVST and in Italy the survey demonstrated that most were satisfied with testing for HIV using a self-test. In both surveys, most participants knew you could test for HIV using a self-test. However, both surveys highlighted issues that may hinder the wide scale uptake of HIVST in the respective countries.

In the Lithuanian survey, most participants reported that they would likely use an HIVST in the future. However, preference for future testing was fairly evenly split between being tested by HCW, using a self-test or not sure. This highlights that HIVST should not be the only option available and individuals should be given a choice. Participants also cited concerns over the price of the test, which was the most common reason why they would not to choose an HIVST; in Lithuania only 15% of participants were willing to pay the current cost for an HIVST. This concern was also expressed by the Italian participants, which indicates the need to offer self-tests at the lowest possible price.

Lithuanian participants were concerned that they would not carry out the test correctly; these findings were also mirrored in Italy, where nevertheless most participants reported being satisfied with the experience. CHWs reported that one third of Italian participants failed one or more steps while carrying out the tests: some of them found it difficult to carry out the test as they did not understand the instructions provided in the leaflet, despite most having a high level of education. This corresponds with another study among people who had recently been diagnosed with HIV after having used an HIVST, where nearly 30% (4/14) had difficulties completing the test or had an indeterminate test result [19]. This indicates the need to provide thorough information on how to take the test and to ensure that it is tailored to the local country’s context. It is also crucial to provide self-test kits users with appropriate resources. In fact, both surveys found that participants would like information about how to obtain support from relevant organisations. These organisations could help by providing assistance while performing the test, post-test counseling and linkage to confirmatory testing in case of a reactive HIVST result. This has also been found in other studies where participants were concerned about not being able to access support if they had a reactive result [20]. Further to this, participants also wanted to test for other infections at the same time and the development of Hepatitis C virus self-test this could help increase uptake of HIVST.

In Italy, participants also reported difficulties in obtaining an adequate amount of blood to complete the test. Currently, in HIVST most commonly available in Italy, only one lancet is provided and if users fail to collect the right amount of blood (e.g. due to vasoconstriction), they would not be able to complete the test. This could be overcome by providing more lancets in the testing kits, to allow users to complete the fundamental step of blood collection. The benefit of collecting information from both the user and the CHW allows us to understand the experience by the user and whether the test was successfully completed.

In both countries, one of the most common reasons for wanting to use an HIVST in the future was privacy and confidentiality. In Lithuania, HIV is still strongly stigmatised and this may indicate a perceived difficulty in accessing confidential HIV testing; HIVST could help to offer an alternative way for individuals to test. This was also found in a study in England, indicating that HIVST benefits of privacy and confidentiality lower the barriers to testing and help to widen access for MSM and black and other minority ethnic groups [21]. In addition, in Lithuania only HCWs can carry out an HIV test and in Italy a HCW needs to be on site during testing activities [8]. HIVST could also help to remove the barrier of medicalised testing, widening access to key population groups.

Among the limitations, it must be noted that the sample size was small in both countries and participants were from a self-selected sample as they were using NGOs to access HIV testing, which may limit the generalisability of the findings to the wider public and this participation bias may not completely disclose the barriers of other individuals to have access to HIV testing. In Lithuania, most participants were either MSM or reported a risk factor for HIV; although this may bias our findings, these are two key population groups who could benefit from accessing HIVST. Information on HIV risk was not collected in the Italian survey, which could have helped in understanding if opinions about using of HIVST varied by HIV risk. These surveys, which are the first ones of their kind, clearly show the acceptability and feasibility of using HIVST as part of the HIV prevention response.

## Conclusion

In both Italy and Lithuania, HIVST are available but have not yet been fully integrated into the national HIV prevention response. There is a paucity of data about the awareness and usability of HIVST. These two surveys aimed to understand the barriers to the full scale implementation of this testing strategy; in Lithuania it was found that HIVST has good acceptability and is a viable testing option; however, many would not be able to afford to pay for an HIVST at the current cost level. Access to HIVST in Lithuania needs to be widened and initiatives to reduce the cost of the test should be explored. In Italy, most participants reported that HIVST were easy to use; yet CHWs reported that some of them failed a step in the testing process, which could have resulted in a potential erroneous test result. Therefore, the current testing instructions need to be improved to help increase HIVST usability. The findings from both surveys will support the development of design of local testing strategies to increase awareness of HIVST, improve testing instructions and tailor support services for those using HIVST.

## Data Availability

The datasets used and analysed during the current study are available from the corresponding author on reasonable request.

## Appendix

**Appendix 1 – Survey of acceptability of HIV self-testing in Lithuania**

### Age

1. What is your gender?

- Man
- Woman
- Trans woman
- Trans man
- Other, please specify

2. In the past 6 months, have you (*tick all that apply*):

- had sex without a condom with a man
- Had sex without a condom with a woman
- Had more than one sexual partner
- Had Sex with a casual partner
- Had Sex with sex worker
- Had contact (including professional) with the blood or other body fluids of an HIV-positive or suspected person
- Had flu like symptoms (fever, rash, diarrohea, nights sweat, increased lymph nodes in the neck, armpits or groin)
- Had more than one fever in a month/ unexplained fever
- Experienced violence or sexual abuse
- A had sex with a Partner who has been diagnosed with HIVor another STI
- Weight loss without reason
- Have been diagnosed with Tuberculosis/TB
- Have been diagnosed with candidiasis (thrush) of the genitals, mouth, esophagus or throat
- Shared syringes / needles for injecting drugs
- Had an Invasive procedureperformed using non-sterile tools

3. Which is the highest educational level you have completed?

- None or primary education (education up to 12 years of age)
- First level secondary or middle grade vocational training (the level that should be finished at 16 years of age)
- Second level secondary or upper grade vocational training (the level that should be finished at 18 years of age)
- High (College)
- High (University)
- Others: please specify

4. When was your last HIV test?

- Never
- In the last 12 months
- More than 12 months ago

5. If you have tested in the last 12 months, how times have you tested?

- Once
- Twice
- 3–4 times
- More than 4 times

6. Are you aware that HIV self-tests are available?

*HIV self-testing is a process whereby a person who wants to know his or her HIV status collects a specimen, performs a test and interprets the test result in private.*

- Yes
- No

7. If you used a HIV self-test, would you believe the test result?

- Definitely yes
- Probably yes
- Maybe
- Probably not
- Definitely not

8. Would you know what to do if you got a reactive HIV self-test result:

- Definitely yes
- Probably yes
- Maybe
- Probably not
- Definitely not

How do you agree with the following statements?

9. I would prefer to do an HIV test at a clinic or community-based organization, rather than a self-test:

- Strongly agree

### Agree

- Neutral (neither agree nor disagree)
- Disagree
- Strongly disagree

10. How likely would you be to do an HIV self-test in the future?

- Very likely
- Somewhat likely
- Not likely

11. What, if any, are the main reasons you would choose an HIV self-test (*tick all that apply*):

- Convenience
- Privacy and confidentiality
- Does not require a visit to a health facility
- Easy to use
- Immediate results

12. What, if any, are the main reasons you WOULD NOT choose an HIV self-test (*tick all that apply*):

- Cost is too expensive/not free
- Afraid of needles
- Afraid of blood
- Lack of counselling
- Concerned about getting the results alone
- Worried the test is not accurate
- Worried about misinterpreting the test result

13. If you were to use an HIV self-test in the future, how would you prefer to use it?

### Alone

- Accompanied by someone
- Other: please specify

14. How much would you be willing to pay for the HIV self-test,?

- Please specify
- I don’t know/ can’t answer

15. How much could you spent for HIV self-test? (choose one)?

- Up to €5
- Up to €10
- Up to €20
- Up to €30+
- I would not be willing to pay

16. Where would you prefer to obtain an HIV self-test (*tick all that apply*)?

- Chemists or pharmacies (over the counter)
- In hospital clinics
- From community organisations / testing CheckPoints
- Online of pharmacy
- Online not pharmacy
- Vending machines
- Other/ I don’t know

17. What additional features would you like to have with an HIV self-test? (*tick all that apply*)

- Access to 24 h counselling and support (online or telephone)
- Contact details for HIV support organisations
- Tests for other STIs, like syphilis, chlamydia, or gonorrhoea
- Other: please specify

18. If you were be able to get HIV self-test FOR FREE, would this lead you to do HIV test?

- Yes, but pre-test consultation would be good
- Yes, but I would like to gest post-test consultation without what the result will be
- No, − I trust only specialist and clinics, not rapid tests
- No, there is no need
- Other: please specify

19. Please share any final thoughts or comments about the HIV self-test:

## Appendix 2 – Survey of usability of HIV self-testing in Italy – completed by community healthcare worker

1. Did the participant use information sheet?
2. Did the participant correctly remove the cap from testing device and place in the stand?
3. Did the participant disinfect their fingertip with the wipe?
4. Was the participant able to lance finger correctly and form a blood droplet?
5. Was the participant able to get any amount of blood into the testing device?
6. Did the participant push the test device into the stand firmly and hear it snap 3 times?
7. Was the participant able to read the test result without assistance?
  - if yes, how?
8. Did the participant quit the process at any point?
  - if yes, why?
9. Please add any other observations

## Appendix 3 – Survey of usability of HIV self-testing in Italy – completed by survey participant

1. What’s your gender?
2. What is your sexual orientation?
3. What country where you are born in?
4. Which is the highest educational level you have completed?
5. When were you last tested for HIV?
6. Were you aware before today that there was a self-test for HIV?
7. How easy was it to complete the test?
8. How easy was it to read the result?
9. On a scale of one to ten, how satisfied are you with the experience?
10. If you have not been satisfied, could you explain why?
11. How reliable do you think the self-test result is?
12. Would you know what to do if the self-test was positive?
13. Would you prefer to test in a hospital or community organisation instead of a self-test?
14. How likely you are to do a self-test in the future?
15. What are the reasons why you would use a self-test in the future?
16. What are the reasons why you would **not** use a self-test in the future?
17. Where would you prefer to buy a self-test for HIV?
18. What other features would you like to accompany the self-test?
19. Any other comments?

## Appendix 4 – Lithuanian study: Characteristics and preferences for HIV self-testing among Lithuanian respondents (*n* = 138)

**Table:**

		n	%
**Gender**	Male	122	88
	Female	16	12
**Age group**	Under 25	24	17
	25–34	64	46
	35 and older	50	36
**Reported risk for HIV** ^**1**^	Yes	119	86
**Highest education level achieved**	Initial	1	1
	Secondary	10	7
	Professional qualifications	6	4
	Graduate College	22	16
	Graduate University	98	71
	Other	1	1
**Ever tested**	Within in last 12 months	89	65
	More than 12 months ago	25	18
	Never	24	17
**Previous HIV testing history**	Not tested in the last year	49	36
	1	28	20
	2	31	22
	3	37	9
	4+	12	12
**Aware of HIV self-tests**	Yes	103	75
**Would you trust the result?**	Definitely	46	33
	More so	64	46
	Not sure	22	16
	More not	3	4
	Definitely not	1	1
**Would you know what to do if you received a reactive/positive result?**	Definitely	51	37
	More so	40	29
	Not sure	30	22
	More not	9	7
	Definitely not	8	6
**Would you prefer to do a blood test over a self-test?**	Strongly agree	31	22
	Agree	32	23
	Neither agree or disagree	44	32
	Disagree	23	17
	Strongly disagree	8	6
**How likely are you to buy and use a self-test in the future?**	Very likely	44	32
	Somewhat likely	58	42
	Unlikely	36	26
**Reasons why you** **would** **self-test**	Privacy and confidentiality	97	70
	Rapid result	82	59
	Anonymity	69	50
	Convenience	71	51
	Easy to use	51	37
**Reasons why you** **would not** **use a self-test**	Price	81	59
	Test incorrectly	58	42
	Cannot discuss result	50	3
	Interpret incorrectly	36	26
	Fear of testing	27	20
	Fear of needle	16	12
	Fear of blood	4	3
**If you bought a test, how would you like to take it?**	Alone	81	59
	With someone else	42	30
	No opinion	15	11
**How much are you** **willing** **to pay?**	Not willing to pay	4	3
	1–5€	26	19
	6–10€	45	33
	11–20€	42	30
	More than 20€	21	15
**How much are you** **able** **to pay?**	Cannot allocate funds	4	3
	Up to 5€	25	18
	Up to 10€	44	32
	Up to 20€	47	34
	Up to 30€	19	14
**Where would you like to buy a self-test?**	Pharmacy	96	70
	Hospital	13	9
	Non-governmental organisation	53	38
	Online	84	61
	Vending machine	58	42
	Other	2	1
**If able to get a test for free what services would to like?**	Counselling and assistance	72	52
	Contact info for support	93	67
	Test for other infections	94	68
	Specialist consultation before	57	41
	Specialist consultation after	82	59
	Other	12	9

1 – Classified as reporting either unprotected sex with a man/woman, had multiple sex partners, sex with causal partner, sex with a sex worker, contact with a person living with HIV, symptoms of acute HIV infection, fever for more than a month, subjected to sexual abuse and violence, partner has HIV or STI diagnosis, sudden weight loss, have / had TB, have / had candidiasis, shared needles, invasive procedures were produced with non-sterile tools or other symptoms

## Appendix 6 – Italian study: Characteristics and preferences for HIV self-testing among Italian participants (*n* = 28)

**Table:**

		n	%
**Gender**	Male	16	57
	Female	12	43
**Sexual orientation**	Heterosexual	17	61
	Gay	10	36
	Lesbian	1	4
**Country of Birth**	Italy	24	86
	Other - Europe	3	11
	Not reported	1	4
**Age group**	Under 25	2	7
	25–34	16	57
	35 and older	10	36
**Highest education level achieved**	Secondary	12	43
	Graduate University	16	57
**Ever tested**	Within in last 12 months	12	43
	More than 12 months ago	9	32
	Never	7	25
**Aware of HIV self-tests**	Yes	19	68
**How easy was it to perform the self-test?**	Very Easy	3	11
	Easy	9	32
	Not easy but not difficult	10	36
	Difficult	5	18
	Very Difficult	1	4
**How easy was it to read the result?**	Very Easy	15	54
	Easy	12	43
	Not easy but not difficult	0	0
	Difficult	0	0
	Very Difficult	1	4
**On a scale of one to ten, how satisfied are you with the experience?**	1	2	7
	2	0	0
	3	0	0
	4	0	0
	5	4	14
	6	2	7
	7	2	7
	8	4	14
	9	6	21
	10	8	29
**How reliable do you think the self-test result is?**	Definitely reliable	13	46
	Probably reliable	14	50
	Not sure	1	4
**Would you know what to do if you received a reactive/positive result?**	Definitely	10	36
	Probably yes	10	36
	Not sure	3	11
	Probably not	4	14
	Definitely not	1	4
**Would you prefer to do a self-test over a test at a non-governmental organisation?**	Strongly agree	6	21
	Agree	3	11
	Neither agree or disagree	9	32
	Disagree	7	25
	Strongly disagree	3	11
**Would you prefer to do a test at a hospital over a self-test?**	Strongly agree	3	11
	Agree	0	0
	Neither agree or disagree	12	43
	Disagree	10	36
	Strongly disagree	3	11
**How likely are you to buy and use a self-test in the future?**	Very likely	8	29
	Likely	12	43
	Not likely	8	29
**Reasons why you** **would** **self-test**	Privacy	6	21
	Rapid result	19	68
	Easy	7	25
	Convenience	11	39
	Not painful	1	4
**Reasons why you** **would not** **use a self-test**	Price	8	29
	Lack of support / counselling	13	46
	Finding out result alone	9	32
	Interpret incorrectly	1	4
	Reliability of the test	4	14
	Fear of needle	1	4
**If you bought a test, how would you like to take it?**	Alone	13	46
	With someone else	15	54
**How much are you** **willing** **to pay?**	Not willing to pay	2	7
	Up to 5€	1	4
	Up to 10€	11	39
	Up to 20€	9	32
	Up to 30€	5	18
**Where would you like to buy a self-test?**	Pharmacy	21	75
	Hospital	3	11
	Non-governmental organisation	10	36
	Online	7	25
	Vending machine	17	61
	Other	2	7
**What other services would you like to accompany a self-test?**	Counselling	14	50
	Contact info for support organisations	17	61
	Test for other STIs	9	32

## Appendix 7 – Italian study: Observations by community healthcare worker assessing a participant using a HIV self-test (*n* = 28)

**Table:**

	Yes (n)	%
**Did the participant use information sheet?**	27	96
**Did the participant correctly remove the cap from testing device and place in the stand?**	26	93
**Did the participant disinfect their fingertip with the wipe?**	26	93
**Was the participant able to lance finger correctly and form a blood droplet?**	21	75
**Was the participant able to get any amount of blood into the testing device?**	22	79
**Did the participant push the test device into the stand firmly and hear it snap 3 times?**	21	75
**Was the participant able to read the test result without assistance?**	22	79
**Did the participant end the process at any point?**	7	25

## Abbreviations

ART: antiretroviral therapy
CHW: community health worker
EU/EEA: Western European countries
HCW: health care worker
HIVST: HIV self-test
MSM: men who have sex with men
PWID: people who inject drugs
NGO: non-governmental organisation

## Footnotes

1 HIV risk was classified as reporting the following: unprotected sex, sex with; a causal partner, multiple partners, sex worker or a person living with HIV, HIV indicator condition, fever for more than a month, experienced sexual abuse and violence, partner has HIV or STI diagnosis, sudden weight loss, TB infection, candidiasis, shared needles, invasive procedures using non-sterile tools.
